# Identification of *S100A8*-correlated genes for prediction of disease progression in non-muscle invasive bladder cancer

**DOI:** 10.1186/1471-2407-10-21

**Published:** 2010-01-25

**Authors:** Seon-Kyu Kim, Eun-Jung Kim, Sun-Hee Leem, Yun-Sok Ha, Yong-June Kim, Wun-Jae Kim

**Affiliations:** 1 Department of Urology, College of Medicine, Chungbuk National University, Cheongju, Chungbuk, South Korea; 2 BK21 Chungbuk Biomedical Science Center, School of Medicine, Chungbuk National University, Chengju, Chungbuk, South Korea; 3 Department of Biological Science, Dong-A University, Busan, South Korea

## Abstract

**Background:**

*S100 calcium binding protein A8 *(*S100A8*) has been implicated as a prognostic indicator in several types of cancer. However, previous studies are limited in their ability to predict the clinical behavior of the cancer. Here, we sought to identify a molecular signature based on *S100A8 *expression and to assess its usefulness as a prognostic indicator of disease progression in non-muscle invasive bladder cancer (NMIBC).

**Methods:**

We used 103 primary NMIBC specimens for microarray gene expression profiling. The median follow-up period for all patients was 57.6 months (range: 3.2 to 137.0 months). Various statistical methods, including the leave-one-out cross validation method, were applied to identify a gene expression signature able to predict the likelihood of progression. The prognostic value of the gene expression signature was validated in an independent cohort (n = 302).

**Results:**

Kaplan-Meier estimates revealed significant differences in disease progression associated with the expression signature of *S100A8*-correlated genes (log-rank test, *P *< 0.001). Multivariate Cox regression analysis revealed that the expression signature of *S100A8*-correlated genes was a strong predictor of disease progression (hazard ratio = 15.225, 95% confidence interval = 1.746 to 133.52, *P *= 0.014). We validated our results in an independent cohort and confirmed that this signature produced consistent prediction patterns. Finally, gene network analyses of the signature revealed that *S100A8*, *IL1B*, and *S100A9 *could be important mediators of the progression of NMIBC.

**Conclusions:**

The prognostic molecular signature defined by *S100A8*-correlated genes represents a promising diagnostic tool for the identification of NMIBC patients that have a high risk of progression to muscle invasive bladder cancer.

## Background

Non-muscle invasive bladder cancer (NMIBC) is the most common histological subtype of bladder cancer, accounting for approximately 80% of all cases. Approximately 20% of these patients experience disease progression to muscle invasive bladder cancer (MIBC) after treatment, a development that is associated with a very poor prognosis for survival. Conventional histopathological parameters, such as tumor stage or grade, are generally considered to be prognostic factors, and numerous biomarkers have been investigated as prognostic indicators of the likelihood that NMIBC will develop into MIBC [1-6].

Members of the *S100 *family of calcium-binding proteins play essential roles in epithelial tissues and participate in a wide range of cellular processes, including transcription, proliferation, and differentiation [7-11]. At least 16 genes that encode members of the *S100 *family, including the gene for *S100A8*, are clustered on human chromosome 1q21 [12,13], in a region that frequently experiences chromosomal rearrangement during tumor development [14,15]. *S100A8 *is reportedly up-regulated in many cancers, including bladder cancer [16-23], and has been implicated in the regulation of tumor cell proliferation and metastasis [16,24-26].

Although numerous diagnostic markers have been investigated as indicators of the risk of disease progression [1-6], none are able to sufficiently predict the behavior of NMIBC [1-6]. *S100A8 *has been suggested to be a predictive biomarker of bladder cancer outcome in several studies [21-23], however, the regulation of *S100A8 *gene expression and whether the genes associated with its expression provides additional insight into the mechanisms of disease progression or tumor invasion have not been studied. Therefore, we analyzed the expression pattern of *S100A8 *and its correlated genes to assess whether their molecular signature could identify patients with a higher likelihood of disease progression.

## Methods

### Patients and tissue samples

Primary NMIBC tissue samples from 103 consecutive cases of patients with histologically diagnosed transitional cell carcinoma were obtained from Chungbuk National University Hospital. To reduce confounding factors for affecting the analyses, any patients diagnosed with a concomitant carcinoma in situ (CIS) lesion or only CIS lesion were excluded. All tumors were macro-dissected, typically within 15 minutes of surgical resection. Each bladder cancer specimen was confirmed as representative by analysis of adjacent tissue in fresh frozen sections from transurethral resection (TUR) specimens, and then frozen in liquid nitrogen and stored at -80°C until use. The collection and analysis of all samples was approved by the Institutional Review Board of Chungbuk National University, and informed consent was obtained from each subject.

Tumors were staged and graded according to the 2002 TNM classification and the 2004 WHO grading system, respectively [27]. A second TUR was performed 2-4 week after the initial resection when it was incomplete or when a high-grade or T1 tumor was detected [27]. Patients with intermediate- or high-risk NMIBC received one cycle of intravesical BCG [27,28]. All patients were followed and managed according to the standard recommendation for treatment of NMIBC [27-29]. In this study, we defined progression of the disease as an increase in stage from either Ta or T1 to T2 or higher after disease relapse [30].

### RNA extraction, microarray experiments, and data processing

Total RNA was isolated by TRIzol reagent (Life Technologies, NY), according to the manufacturer's protocol. The quality and integrity of the RNA were confirmed by agarose gel electrophoresis and ethidium bromide staining, followed by visual examination under ultraviolet light. Five-hundred nanograms of total RNA were used for labeling hybridization according to the manufacturer's protocols (Illumina HumanWG-6 BeadChip, version 2). Arrays were scanned with an Illumina Bead Array Reader confocal scanner (BeadStation 500GXDW; Illumina, Inc., San Diego, CA) according to the manufacturer's instructions. After scanning, the microarray data were normalized using quantile normalization in the R language environment (version 2.8.1, available at http://www.r-project.org/). Measured gene expression values were log2 transformed and median centered across genes and samples. The full microarray data set is available in the NCBI Gene Expression Omnibus public database under the data series accession number GSE13507.

### Statistical analysis

To classify patients into two groups, we used the median gene expression value of *S100A8 *as the cut-off. Pearson correlation coefficients were calculated to evaluate the association between *S100A8 *and its correlated genes. A hierarchical clustering algorithm, using the uncentered correlation coefficient as the measure of similarity and average linkage clustering, was applied as described in Eisen et al [31]. The Kaplan-Meier method was used to calculate the time to progression, and differences between the times was assessed using log rank statistics. The prognostic value of the *S100A8*-correlated gene signature was determined with multivariate Cox proportional hazard regression models.

To validate the expression signature of *S100A8 *and its correlated genes identified in our original cohort, we applied it to independent microarray data from 302 patients with NMIBC, reported by Dyrskjot et al [32]. For validation of the prognostic value of the molecular signature, we developed prediction models using the compound covariate predictor [33], Bayesian compound covariate predictor [33], linear discriminator analysis [34], nearest centroid classification [34], and support vector machines [35]. The models incorporated genes that were differentially expressed between the two classes using a two-sample t-test. Genes were considered to have statistically significant differences in expression if the *P*-value was less than 0.001. We estimated the prediction error of each model using leave-one-out cross-validation (LOOCV), as described by Simon et al [36]. For each LOOCV training set, the entire model-building procedure was repeated, including the gene selection process. Validation procedure was performed in BRB ArrayTools (version 3.7.1).

To explore the relationships between *S100A8*-correlated genes, we examined functional associations among the genes and generated gene networks based on whether they had more interconnected genes than would be expected to occur by chance. The significance of each network was estimated using the scoring system provided by the Ingenuity Pathway Analysis Tool (version 7.5). The scores were determined by the number of differentially expressed genes within each of the networks and the strength of the associations among the network members.

## Results

### Baseline characteristics

Table 1 details the baseline characteristics of the 103 primary NMIBC patients. The median age was 66.0 years (range: 24 to 88 years), and the median follow-up period after surgery was 57.6 months (range: 3.2 to 137.0 months). During the follow-up period, 11 of the 103 (10.7%) experienced disease progression.

**Table 1:** Baseline characteristics of primary non-muscle invasive bladder cancer patients

Variable	No. of patients (%)
Sex	
Male	87 (84.5)
Female	16 (15.5)
Grade	
Low	86 (83.5)
High	17 (16.5)
Stage	
Ta	23 (22.3)
T1	80 (77.7)
Progression	
No	92 (89.3)
Yes	11 (10.7)

### Prognostic utility of *S100A8*-correlated genes

**Figure 1:**
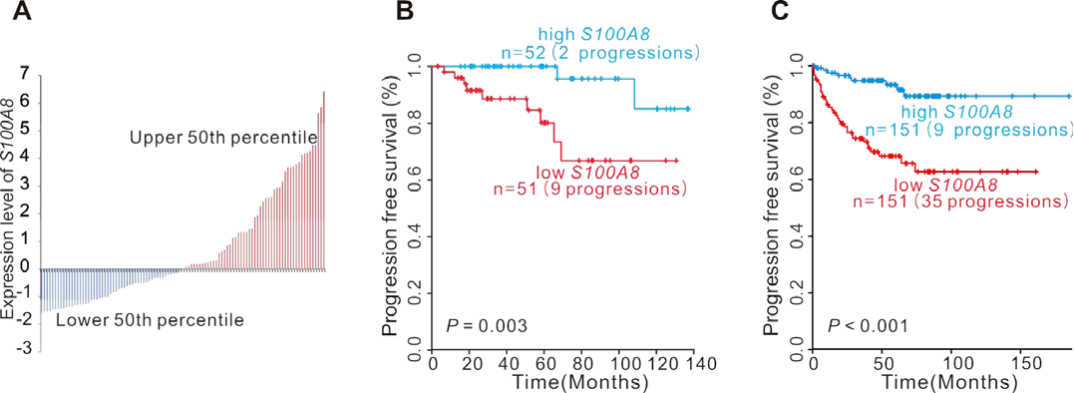
**Expression of *S100A8 *and progression of non-muscle invasive bladder cancer**. A: Expression of *S100A8 *in 103 patients from the original cohort. B: Kaplan-Meier curves showing time to progression in the original cohort. C: Kaplan-Meier curves showing time to progression in the independent European cohort.

We analyzed 103 NMIBC samples and divided them into two groups according to the expression level of *S100A8*. The frequency of progression was significantly higher in the group with *S100A8 *expression levels in the upper 50th percentile than in the group with *S100A8 *expression levels in the lower 50th percentile (log-rank test, *P *= 0.003; Figure 1-A and 1-B).

**Figure 2:**
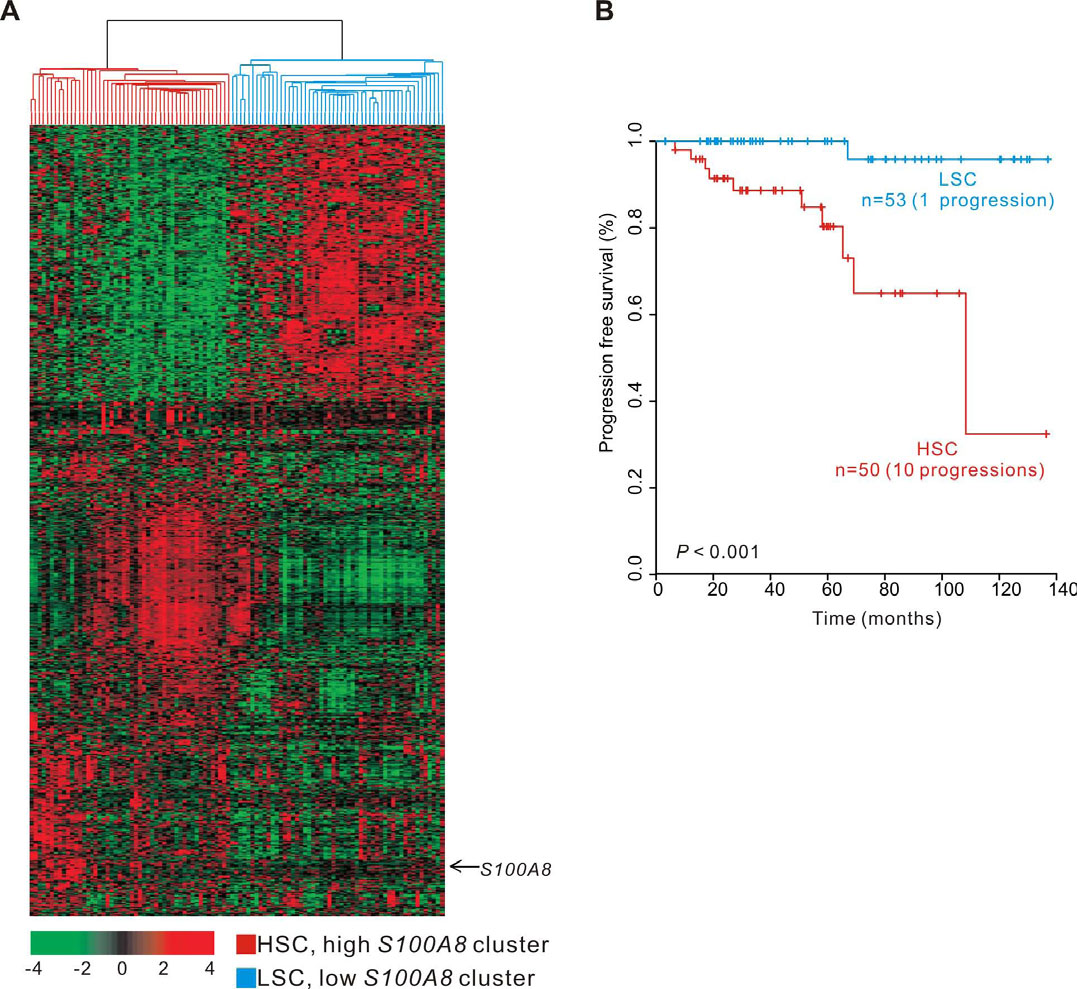
**Gene expression pattern of *S100A8*-correlated genes and progression of two clusters**. A: Gene expression patterns of *S100A8 *and its correlated genes. A total of 1,015 genes whose expression patterns are highly correlated with *S100A8 *were selected for cluster analysis (Pearson correlation test, *P *< 0.001, *r *< -0.3 or *r *> 0.3). Patients were divided into two groups: high *S100A8 *cluster (HSC) and low *S100A8 *cluster (LSC). B: Kaplan-Meier curves showing time to progression. The progression rate of HSC patients was significantly higher than that of LSC patients (log-rank test, *P *< 0.001).

We next sought to identify a gene expression signature that directly correlated with *S100A8 *expression levels during disease progression and then use that signature to predict the likelihood of tumor progression. We identified 1,015 genes whose change in expression correlated with *S100A8 *expression (Pearson correlation test, *P *< 0.001, *r *< -0.3 or *r *> 0.3). Based on hierarchical clustering analysis of the expression patterns of these genes, we divided the NMIBC samples into two groups: high *S100A8 *cluster (HSC) and low *S100A8 *cluster (LSC). The progression rate of HSC patients was significantly higher than that of the LSC patients (log-rank test, *P *< 0.001; Figure 2).

To evaluate the prognostic efficacy of the newly identified signature, we applied multivariate Cox regression analysis to the signature and known clinical and pathologic prognostic factors for NMIBC (Table 2). This analysis revealed that the molecular signature of *S100A8*-correlated genes (hazard ratio 15.225, 95% confidence interval = 1.746 to 133.52, *P *= 0.014) was the only strong predicator of bladder cancer progression.

**Table 2:** Multivariate Cox regression analysis for prediction of disease progression

Variable	Progression	
	**HR (95% CI)**	***P*-value**

Stage (Ta vs. T1)	0.258 (0.032 - 2.083)	0.204
Grade (low vs. high)	2.257 (0.445 - 11.449)	0.326
Number of tumors		
Single	Reference	-
2 to 7	1.684 (0.314 - 9.027)	0.543
>8	4.544 (0.624 - 32.141)	0.129
Size (> 3 cm vs. ≤ 3 cm)	1.933 (0.397 - 9.411)	0.414
Intravesical therapy (Yes vs. No)	2.141 (0.391 - 11.715)	0.38
*S100A8*-correlated gene signature (HSC vs. LSC)	15.225 (1.736 - 133.52)	0.014

Abbreviations: HR, hazards ratio; CI, confidence interval

### Validation of the signature in an independent cohort

We next sought to validate our findings by using gene expression data from an independent cohort of European patients with bladder cancer [32]. Patients with NMIBC from this cohort (n = 302) were divided into two groups according to the expression level of *S100A8*. Consistent with our results, progression of NMIBC was significantly higher in patients with *S100A8 *expression levels in the upper 50th percentile than in those with *S100A8 *expression levels in the lower 50th percentile (log-rank test, *P *< 0.001; Figure 1-C).

**Figure 3:**
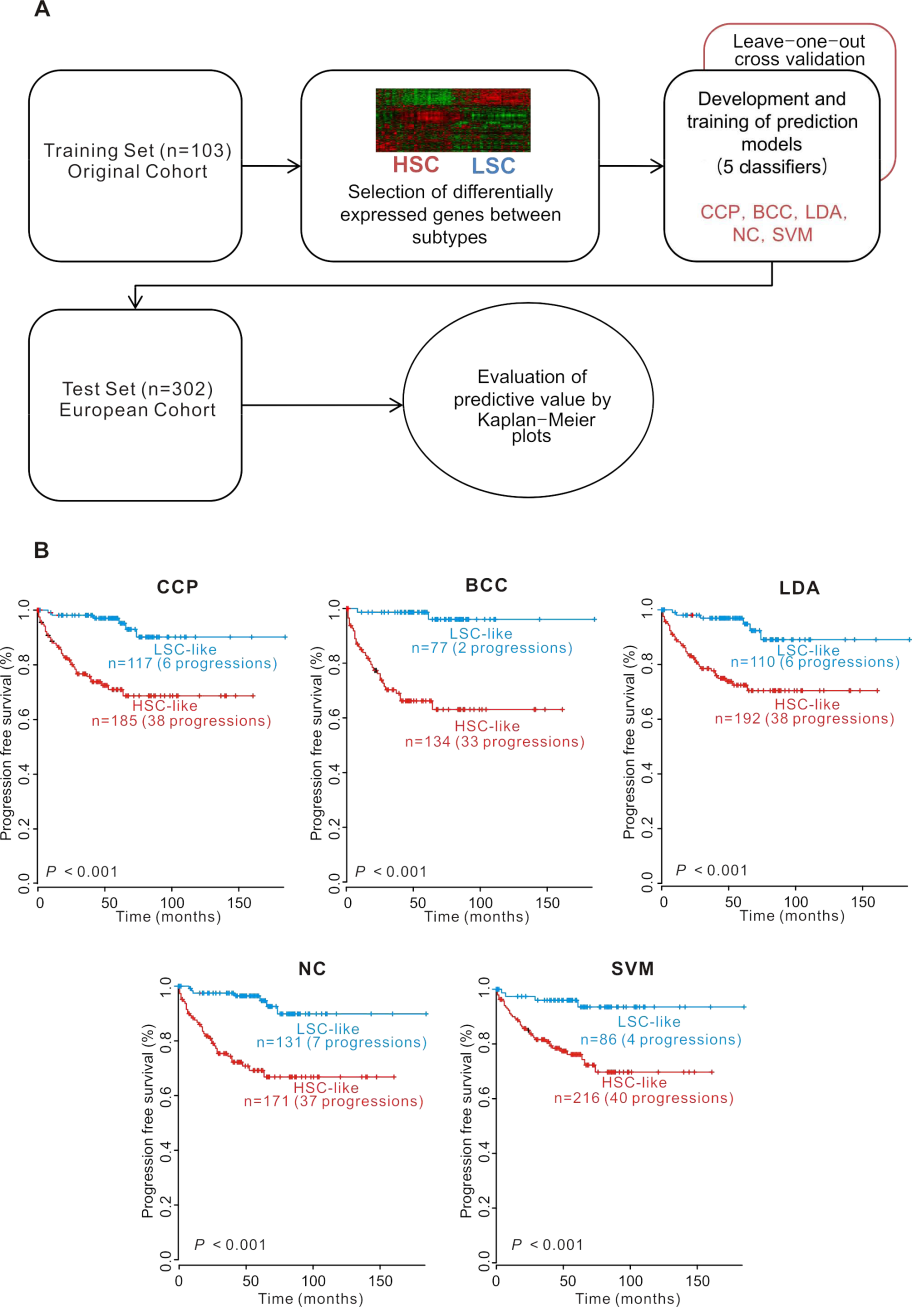
**Independent validation of the prognostic value of the signature**. A: The validation strategy used for the construction of prediction models and the evaluation of predicted outcomes based on gene expression signature. B: Kaplan-Meier plots of progression of NMIBC patients from an independent European cohort predicted by compound covariate predictor (CCP), Bayesian compound covariate predictor (BCC), linear discriminator analysis (LDA), nearest centroid classification (NC), and support vector machines (SVM).

We also validated the association of the *S100A8*-correlated gene signature with disease progression in the European cohort. To overcome the peculiarities of any one particular prediction algorithm, we applied five different statistical methods to test the accuracy of our signature-based prediction of disease progression (Figure 3). We identified the genes with the greatest difference in expression level between the HSC and LSC subgroups in our original cohort (the training set). These genes were pooled to form a series of classifiers able to estimate the probability that a particular bladder cancer sample belonged to the HSC or LSC subgroup. The number of genes in the classifier set was optimized to minimize misclassification during LOOCV of the tumors in the training set. The performance of each prediction model is illustrated in Table 3. When applied to the European cohort (the test set), all five models produced consistent prediction patterns. Kaplan-Meier estimations in the test set revealed significant differences in the risk of progression between patients in subgroups HSC and LSC (Figure 3-B).

**Table 3:** Performance of prediction models

CCP				
**Class**	**Sensitivity**	**Specificity**	**PPV**	**NPV**

HSC	0.92	0.943	0.939	0.926
LSC	0.943	0.92	0.926	0.939

**BCC**				

**Class**	**Sensitivity**	**Specificity**	**PPV**	**NPV**

HSC	0.88	0.887	0.88	0.887
LSC	0.887	0.88	0.887	0.88

**LDA**				

**Class**	**Sensitivity**	**Specificity**	**PPV**	**NPV**

HSC	0.9	0.943	0.938	0.909
LSC	0.943	0.9	0.909	0.938

**NC**				

**Class**	**Sensitivity**	**Specificity**	**PPV**	**NPV**

HSC	0.9	0.962	0.957	0.911
LSC	0.962	0.9	0.911	0.957

**SVM**				

**Class**	**Sensitivity**	**Specificity**	**PPV**	**NPV**

HSC	0.96	0.962	0.96	0.962
LSC	0.962	0.96	0.962	0.96

Sensitivity and specificity of compound covariate predictor (CCP), Bayesian compound covariate predictor (BCC), linear discriminator analysis (LDA), nearest centroid classification (NC), and support vector machines (SVM). Sensitivity is the probability for a class A sample to be correctly predicted as class A. Specificity is the probability for a non class A sample to be correctly predicted as non-A. Positive Predictive Value (PPV) is the probability that a sample predicted as class A actually belongs to class A. Negative Predictive Value (NPV) is the probability that a sample predicted as non class A actually does not belong to class A.

For some class high *S100A8 *cluster (HSC), if n11 = number of class HSC samples predicted as HSC, n12 = number of class HSC samples predicted as low *S100A8 *cluster (LSC), n21 = number of LSC samples predicted as HSC, and n22 = number of LSC samples predicted as LSC, then the following parameters can characterize performance of classifiers: Sensitivity = n11/(n11+n12), Specificity = n22/(n21+n22), PPV = n11/(n11+n21), and NPV = n22/(n12+n22).

### Biological insights into the signature for disease progression

**Figure 4:**
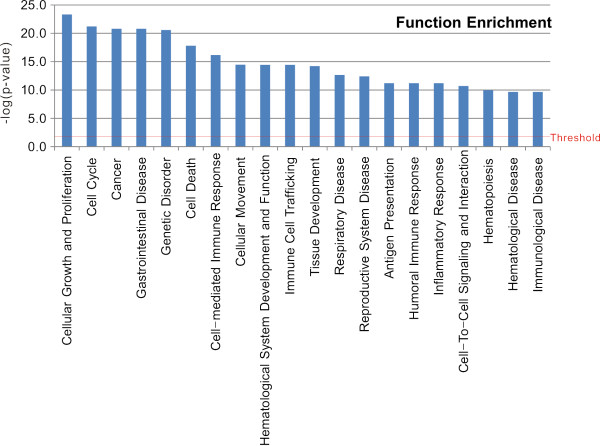
**Functional classification of *S100A8*-correlated genes**. Classification enrichment was determined using Ingenuity Pathway Analysis software. The threshold of significance was -log (*P *= 0.05).

To identify the predominant signalling networks active in the advancement from NMIBC to MIBC, gene network analysis of the 1,015 genes featured in the progression signature (Figure 2) was carried out using Ingenuity™ Pathways Analysis software. Of the 1,015 genes, 768 were mapped to gene networks defined by this tool. This analysis revealed a series of putative networks and associated functional categories. The 10 putative networks with highest scores are listed in Additional file 1 and their associated functions are illustrated in Figure 4.

As expected, genes involved in cellular growth and proliferation, cell cycle, cancer, and cell death were enriched, providing confidence in our results. We also found that genes involved in cell-mediated immune responses, immune cell trafficking, humoral immune responses, inflammatory responses, and immunological disease were significantly enriched (Figure 4).

**Figure 5:**
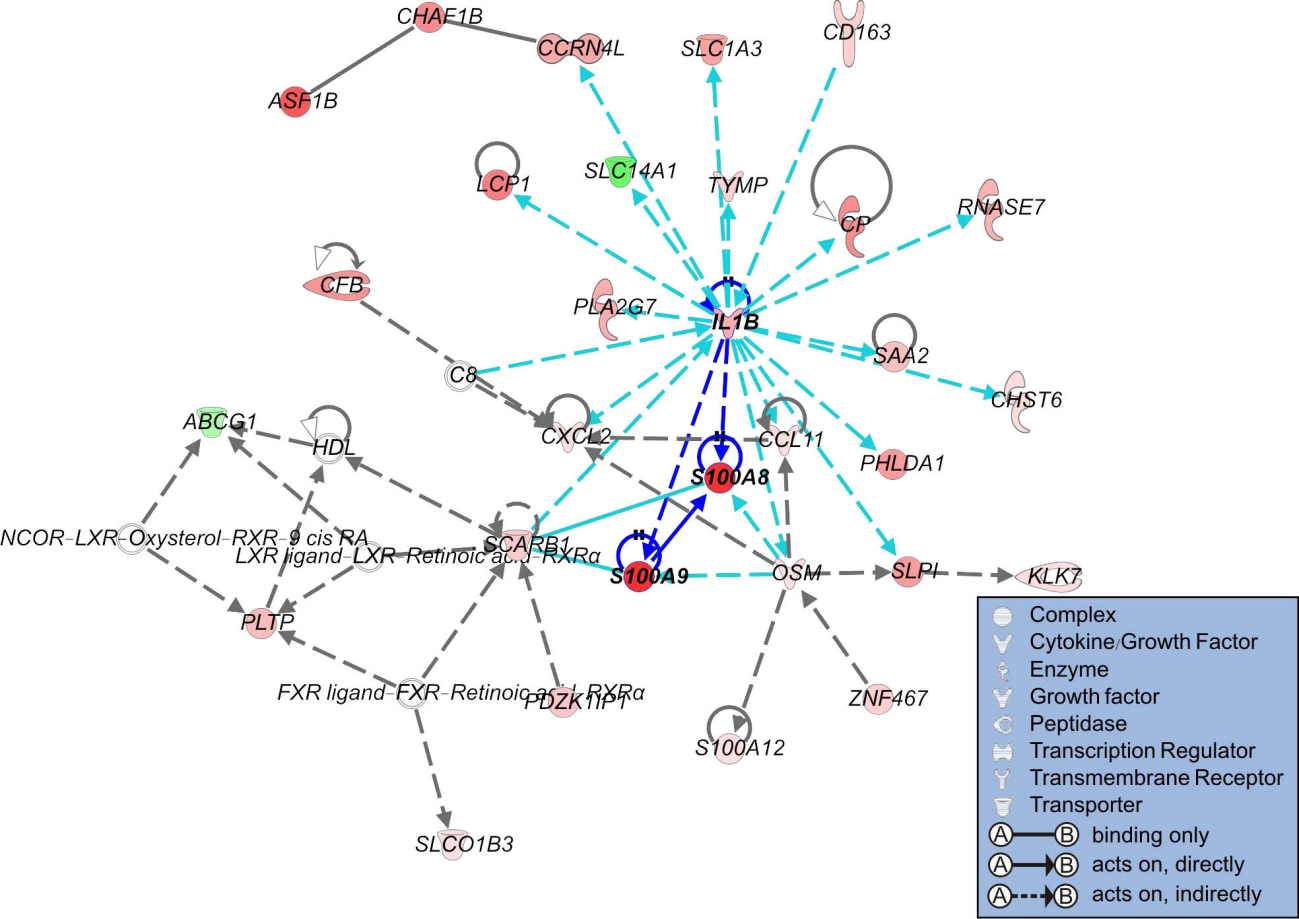
**Gene networks enriched with genes associated with *S100A8***. Gene networks of 1,015 genes that highly correlated with *S100A8*. Up- and down-regulated genes in the high *S100A8 *cluster (HSC) group are indicated in red and green, respectively. The intensity of color is indicative of the degree of over- or under-expression. Genes without highlighted color are not part of the progression signature but are associated with the regulated genes. Each line and arrow represents functional and physical interactions between the genes and the direction of regulation reported in the literature.

**Figure 6:**
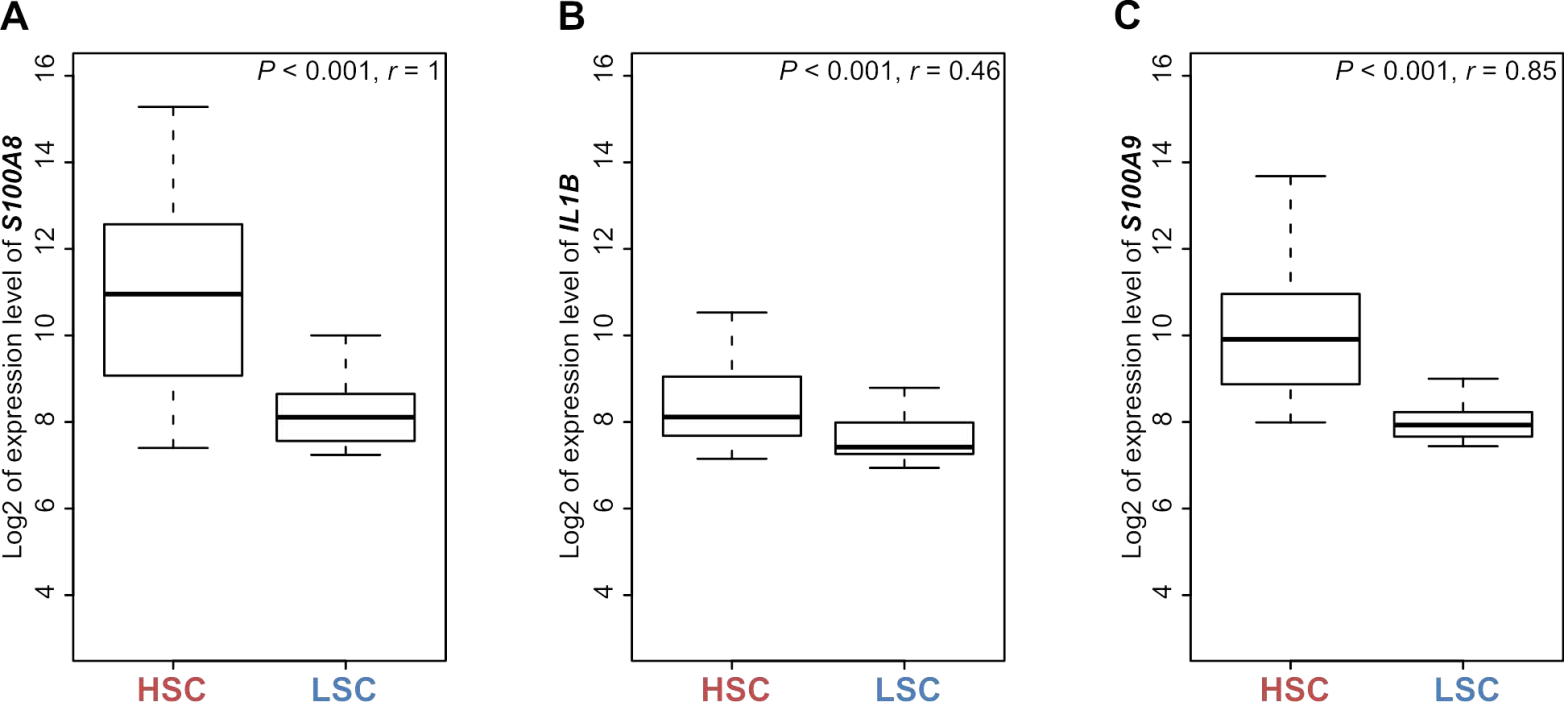
**Comparison of expression levels between high *S100A8 *cluster (HSC) and low *S100A8 *cluster (LSC) patients**. Two group box plot comparing expression levels of *S100A8 *(A), *IL1B *(B), and *S100A9 *(C) in HSC and LSC patients. *P*-value was obtained by two-sample t-test between HSC and LSC. The value of *r *indicates the correlation coefficient value of the gene compared with *S100A8*.

Interestingly, among the top 10 putative networks, we observed functional connectivity between *S100A8 *and *IL1B *in the third network, in which *S100A8 *is a downstream effector of *IL1B *[37] (Figure 5). The expression level of *IL1B *was significantly higher in the HSC than in the LSC subgroup (two-sample t-test, *P *< 0.001; Figure 6-B), indicating its potential role in the progression of NMIBC to MIBC. We also found functional relationship between *S100A8 *and *S100A9*, in which *S100A9 *is a direct binding partner of *S100A8 *(Figure 5). *S100A9 *was more highly expressed in the HSC than in the LSC subgroup (two-sample t-test, *P *< 0.001; Figure 6-C).

## Discussion

The data presented in this study demonstrates that the expression signature of *S100A8*-correlated genes is able to predict the likelihood of bladder cancer progression. The validity of this signature as a prognostic indicator was confirmed by analysis of 302 cancers from an independent European cohort. In addition, based on the results of gene network analysis, we have identified a putative mechanism that may be responsible for disease progression.

Although considerable effort has been devoted to the establishment of a prognostic model of NMIBC that can provide information concerning survival and treatment options at diagnosis [1-6], the ability to predict the course of disease progression for patients with bladder cancer remains a major clinical challenge. A number of the molecular markers that have been identified to date have been explored as potential predictors of disease progression. Some of these, such as *p53*, have been suggested to be independent markers, while others do not appear to be effective as prognostic indicators [4-6]. Thus, there is a crucial need for methods capable of identifying patients with NMIBC that is likely to develop into MIBC. In the present study, we developed a method to predict the progression of primary NMIBC based on a gene expression signature. We showed that our method has strong predictive value through multivariate regression analysis and a validation study in an independent cohort. These data underscore the effectiveness of this molecular signature as a prognostic indicator in NMIBC, and suggest that this signature could be clinically useful.

The accumulation of recent reports that document the deregulated expression of calcium-binding proteins of the *S100 *family in a variety of human cancers implies that *S100 *proteins are required for neoplastic or metastatic transformation of tumor cells [26]. Previous observations that *S100A8 *expression is enhanced in bladder cancer suggest a role for this protein in the progression of bladder cancer [21-23]. The data obtained from the current study demonstrated that upregulation of *S100A8 *expression was strongly associated with disease progression. This result substantiates the involvement of *S100A8 *during the progression of NMIBC.

Expression of *S100A8 *is not the only indicator of *S100A8 *activity, because it is regulated by many different mechanisms. The identification of stable and reliable human gene-to-gene relationships is an essential step towards unraveling the interactions and functional correlations between human genes [38]. Therefore, we performed gene network analysis to identify the association of *S100A8*-correlated genes with progression of NMIBC. Strikingly, expression of both *S100A8 *and its correlated genes were strong predictors of the progression of bladder cancer (Figures 1 and 2). This finding was further supported by multivariate analysis, which showed that the molecular signature of *S100A8*-correlated genes was a strong predictor of cancer progression-free survival (Table 2). This result suggests that the *S100A8*-correlated gene signature retains its prognostic relevance even after additional pathological prognostic features have been taken into account.

The standard strategy for evaluating the accuracy of classification methods is application of a training-validation approach, in which a training set is utilized to identify the molecular signature and a validation set is used to estimate the degree of reliability. For the validation assay, a large sample size is needed before expression profiling can be utilized in a clinical setting [39]. In the current study, the accuracy of a predictive *S100A8*-correlated gene signature was tested using five independent algorithms with gene expression data from a large independent European cohort (Figure 3). This result demonstrated not only a strong association between gene expression patterns and progression, but also provided strong evidence of the reliability of the prediction.

Based on an analysis of the *S100A8*-correlated genes in the context of gene networks, we identified a putative role for *S100A8 *in disease progression of bladder cancer. Because *S100A8 *is the downstream target of *IL1B *[37], our results strongly support the potential involvement of *IL1B *in tumor progression. Recent studies indicate that *IL1B *is associated with tumor invasiveness and metastasis [40,41]. Many of the satellite genes associated with *IL1B *(i.e., *CXCL2*, *PLA2G7*, *CCL11*, *S100A9*, *CD163*, *RNASE7*, and *OSM*) (Figure 5) participate in inflammatory and immune responses, which are the best known activities of *IL1B*. Previous studies have demonstrated that *IL1B *activates inflammation that promotes tumor invasiveness [40]. It was reported previously that *S100A9 *is up-regulated in conjunction with *S100A8 *in many cancers, including gastric cancer [20], prostate cancer [16,17], and colorectal cancer [18,19]. Both *S100A8 *and *S100A9 *have been implicated in the regulation of cell proliferation [16,24] and metastatic processes [25]. Thus, *S100A9 *may prove a valuable target for prevention of the migration of tumor cells to pre-metastatic sites [26].

Our results demonstrate that an expression signature consisting of *S100A8 *and its correlated genes can be a reliable prognostic indicator of progression in NMIBC, independent of traditional pathologic prognostic parameters. The use of this signature as a predictive indicator could potentially enable the prognosis of heterogeneous NMIBC patients to be determined at diagnosis, which would allow for individualized treatment and evaluation.

## Conclusions

We describe a prognostic molecular signature based on gene expression of *S100A8*-correlated genes that can identify patients with high-risk NMIBC that is likely to develop into MIBC. Identification of patients with high-risk NMIBC may improve the effectiveness of currently available treatments and provide opportunities for the development of new treatment modalities.

## Abbreviations

NMIBC: (non-muscle invasive bladder cancer); MIBC: (muscle invasive bladder cancer); TUR: (transurethral resection); LOOCV: (leave-one-out cross validation); HSC: (high *S100A8 *cluster); LSC: (low *S100A8 *cluster).

## Competing interests

The authors declare that they have no competing interests.

## Authors' contributions

SKK conceived of the study, carried out the statistical analyses, and drafted the manuscript. EJK and SHL performed microarray preparation, sample selection, and RNA isolation. YSH investigated the clinical records of the considered patients and contributed to the interpretation of the results. YJK performed the statistical analyses, contributed to the interpretation of the results, and helped to draft the manuscript. WJK designed the study concept, interpreted the results and approved the final manuscript. All authors have read and approved the final manuscript.

## Pre-publication history

The pre-publication history for this paper can be accessed here:

http://www.biomedcentral.com/1471-2407/10/21/prepub

## Supplementary Material

Additional file 1 **The list of gene networks**. Top10 list of gene networks constructed by Ingenuity™ Pathway Analysis. Click here for file
